# Lipidomic and Antioxidant Response to Grape Seed, Corn and Coconut Oils in Healthy Wistar Rats

**DOI:** 10.3390/nu9010082

**Published:** 2017-01-20

**Authors:** Abraham Wall-Medrano, Laura A. de la Rosa, Alma A. Vázquez-Flores, Gilberto Mercado-Mercado, Rogelio González-Arellanes, José A. López-Díaz, Aarón F. González-Córdova, Gustavo A. González-Aguilar, Belinda Vallejo-Cordoba, Francisco J. Molina-Corral

**Affiliations:** 1 Instituto de Ciencias Biomédicas, Universidad Autónoma de Ciudad Juárez, Anillo Envolvente del PRONAF y Estocolmo s/n, Ciudad Juárez 32310, Chihuahua, Mexico; alma.vazquez@uacj.mx (A.A.V.-F.); gil_4783@yahoo.com.mx (G.M.-M.); rga.nut@gmail.com (R.G.-A.); joslopez@uacj.mx (J.A.L.-D.); 2 Centro de Investigación en Alimentación y Desarrollo, AC (Unidad Hermosillo), Coordinación de Tecnología de Alimentos de Origen Animal (DTAOA), Carretera a la Victoria km. 0.6, AP 1735, Hermosillo 83000, Sonora, Mexico; aaronglz@ciad.mx (A.F.G.-C.); vallejo@ciad.mx (B.V.-C.); 3 Centro de Investigación en Alimentación y Desarrollo, AC (Unidad Hermosillo), Tecnología de Alimentos de Origen Vegetal (DTAOV), Carretera a la Victoria km. 0.6, AP 1735, Hermosillo 83000, Sonora, Mexico; gustavo@ciad.mx; 4 Centro de Investigación en Alimentación y Desarrollo, AC. (Unidad Cuauhtémoc), Laboratorio de Tecnología de Alimentos de Origen Vegetal y Toxicología, Ave. Río Conchos s/n, Parque Industrial, AP 781, Cuauhtémoc 31570, Chihuahua, Mexico; javiermolina@ciad.mx

**Keywords:** specialty oil, fatty acids, grapeseed oil, antioxidant, HDL, coconut oil, corn oil, lipoproteins, phytosterols

## Abstract

Specialty oils differ in fatty acid, phytosterol and antioxidant content, impacting their benefits for cardiovascular health. The lipid (fatty acid, phytosterol) and antioxidant (total phenolics, radical scavenging capacity) profiles of grapeseed (GSO), corn (CO) and coconut (CNO) oils and their physiological (triacylglycerides, total and HDL-cholesterol and antioxidant capacity (FRAP) in serum and fatty acid and phytosterol hepatic deposition) and genomic (HL, LCAT, ApoA-1 and SR-BP1 mRNA hepatic levels) responses after their sub-chronic intake (10% diet for 28 days) was examined in healthy albino rats. Fatty acid, phytosterol and antioxidant profiles differed between oils (*p* ≤ 0.01). Serum and hepatic triacylglycerides and total cholesterol increased (*p* ≤ 0.01); serum HDL-Cholesterol decreased (*p* < 0.05); but serum FRAP did not differ (*p* > 0.05) in CNO-fed rats as compared to CO or GSO groups. Hepatic phytosterol deposition was higher (+2.2 mg/g; *p* ≤ 0.001) in CO- than GSO-fed rats, but their fatty acid deposition was similar. All but ApoA-1 mRNA level increased in GSO-fed rats as compared to other groups (*p* ≤ 0.01). Hepatic fatty acid handling, but not antioxidant response, nor hepatic phytosterol deposition, could be related to a more efficient reverse-cholesterol transport in GSO-fed rats as compared to CO or CNO.

## 1. Introduction

Cardiovascular disease (CVD) continues to be the leading cause of mortality worldwide. It accounts for 17.3 million deaths per year, and it is expected to grow steadily by 2030 [1,2]. The World Health Organization estimates that failure to implement prevention and therapy strategies for CVD could result in an expense of $47 trillion dollars in the next 25 years, a cost that will be borne more heavily by low and middle income countries in which atherosclerotic CVD is the reason for about 50% of all deaths [1]. CVD includes several illnesses, such as coronary heart disease, atherosclerosis and stroke, in which many environmental and genetic factors concur [3,4]. Fortunately, small dietary and behavioral changes may result in a significant reduction of several CVD risk factors [5].

The association of specific rather than total lipid intake on the risk for CVD has been documented for many years. It is well known that the intake of saturated fatty acids (SFA) increases blood triacylglycerides (TAG), total (TC), very low (VLDL-C) and low density lipoprotein (LDL-C) cholesterol, leading to a higher risk for atherosclerosis and coronary heart disease [6,7]. Despite the latter, in silico and wet lab studies have revealed that lauric acid (C_12:0_) from coconut oil (CNO) may exert a TC-lowering effect [8]. However, a low SFA intake without any other dietary modification reduces not only the atherogenic (LDL-C), but also the cardio-protective (HDL-C) cholesterol [9]; the partial replacement of SFA with either mono- (MUFA) or poly- (PUFA) unsaturated fatty acids (FA) reduces the risk for myocardial infarctions and stroke among high-risk persons [10,11]; and the cardio protective effects of omega 3 (*n*-3) PUFA, particularly α-linolenic acid (C_18:3_), have been extensively documented [12,13,14].

More recently, phytosterols (PST) and antioxidants (AOX), commonly found in substantial amounts in specialty oils, such as grape seed (GSO) [15], corn (CO) [16] and pecan nut (PNO) [17] oils, have been associated with a lower risk for oxidative stress, inflammation and dyslipidemia that in concerted action affect the endothelial integrity [18,19]; in fact, synergistic effects between these molecules may result in an even better lipidomic effect [20]. However, the aforementioned edible oils and others differ in their FA, PST and AOX profile, which may impact differently their benefits for cardiovascular health [21]. Therefore, the aim of this study was to evaluate the lipid and AOX profile of corn oil (CO), GSO and CNO and their physiological and genomic effect (reverse-cholesterol transport) after a sub-chronic intake (28 days) in healthy Wistar rats.

## 2. Materials and Methods

### 2.1. Edible Oils and Chemicals

Commercial grapeseed oil (GSO; Olitalia, Italy) and coconut oil (CNO; Everland Natural Foods, Burnaby, BC, Canada) were imported from Canada, and corn oil (CO; Mazola, ACH Food Companies Inc., Cordoba, TN. USA) was obtained from the local market, wrapped in dark plastic bags and transported to the laboratory under refrigeration (4 °C). Casein (ANRC, 95% protein), AIN-93-vitamin mix, AIN-93G-mineral mix, cellulose and DL-methionine were purchased from Bioserv, Inc. (Frenchtown, NJ, USA).

All other food-grade ingredients used to prepare the experimental diets were purchased in the local market. Folin–Ciocalteu reagent (FCR), trolox (6-hydroxy-2,5,7,8-tetramethylchroman-2-carboxylic acid), DPPH (2,2-diphenyl-1-picrylhydrazyl), ABTS^2−^ (2,2′-azinobis-(3-ethylbenzothiazoline-6-sulphonate), gallic acid, quercetin and choline chloride (99% pure, 74.6% choline) were purchased from Sigma Chemical Co. (St. Louis, MO, USA), while standards of fatty acid methyl esters (Supelco^®^ 37 Component FAME Mix) and sterols (campesterol, ergosterol, stigmasterol and β-Sitosterol) were from Supelco, USA (St. Louis, MO, USA). Kits for lipid determination were from Stanbio Laboratory (Boerne, TX, USA). Unless otherwise specified, all ACS-grade solvents were purchased from JT Baker (Mexico City, Mexico) or Fisher Scientific (Houston, TX, USA). All reagents and chemicals used in gene expression analyses were from Sigma-Aldrich (St. Luis, MO, USA) or Promega (Madison, WI, USA).

### 2.2. Fatty Acid and Phytosterol Profile of Edible Oils

The fatty acid (FA) composition of GSO, CO or CNO was analyzed by GC-MS as fatty acid methyl esters (FAMEs) [22], following the method proposed by Villa-Rodríguez et al. [23] in a GC-MS (VARIAN Saturn 2100D) equipped with a CP7420 column (100 m, 0.25 mm i.d.) using helium ultra-high purity grade (1 mL/min) as the carrier gas. Operating conditions were: oven (T (°C)/time (min)/rate (°C/min)): 160/4/20 and 198/42/1, injector (EFC Type 1) and detector temperatures were 250 °C and 180 °C, respectively. The mass spectrometer was operated in the electron impact (EI) mode at 70 eV in the scan range of 40–500 *m*/*z*. FAMEs were identified by comparing the peak’s retention against commercial standards (Supelco 37 FAME Mix; Sigma Chemical Co., St. Louis, MO, USA) and by comparing the respective ion chromatograms with those reported in the NIST 2008 library (NIST/EPA/NIH Mass Spectral Library, Version 2.0). Phytosterol (PST) composition in all three oils was evaluated by direct saponification (KOH 0.5 M) and capillary gas chromatography following the method proposed by Fleteouris et al. [24] in a 6890 GC System Gas Chromatograph (Hewlett-Packard Development Company, L.P., Houston, TX, USA) equipped with a SP™-2560 Capillary GC Column (L × I.D. 100 m × 0.25 mm, d_f_ 0.20 μm;) using helium Ultra High Purity grade (1 mL/min) as the carrier gas.

### 2.3. Total Phenolic Compounds and Antioxidant Capacity

Total phenolic compounds (TP) in edible oils were quantified with the FCR [25] at 665 nm, and results were expressed as milligrams of gallic acid equivalents (GAE) per 100 g of edible oil (mg GAE/100 g). The radical scavenging capacity (RSC) of edible oils was tested against DPPH (518 nm) and ABTS^2−^ (734 nm) radicals, as suggested by Brand-Williams et al. [26] and Re et al. [27], respectively; values were expressed as millimoles of trolox equivalents per liter (mM TE). Lastly, serum (1:1000 dilution) total antioxidant capacity (TAC) was assayed by the ferric reducing/antioxidant power (FRAP) assay at 595 nm using FeSO_4_ (in water) as the standard as previously reported [28]; values are expressed as mM TE.

### 2.4. Bioassay Protocol

The experiment was conducted in pathogen-free male Wistar rats (300 g) obtained from the Universidad Autónoma de Ciudad Juárez (UACJ) animal care facility. Rats were randomly assigned to three groups (*n* = 6 each) and fed ad libitum with one of three iso-energetic diets (399 kcal/100 g diet: 23%, 20% and 57% energy from fat, protein and carbohydrates, respectively) (Table 1).

This formulation was initially based on the nutrient requirements to sustain an adequate growth in young rats [29], mimicking the AIN-93G rodent diet, but differing on the amount (10 g instead of 5 g/100 g), caloric contribution (23% instead of 17%) and lipid source (GSO, CO and CNO instead of soybean oil). After 1 week of acclimatization and during the 4 weeks of experimental treatment, rats were housed individually in metabolic cages under controlled environmental conditions (22 ± 2 °C, relative humidity 45%–60%, 12-h light to dark cycles). Animals and residual diets were weighed every other day, and the feed efficiency ratio (FER = weight gain (g)/diet consumed (g)) was calculated. At the end of the study, animals were sacrificed under anesthesia (tiletamine-zolazepam (Zoletil^®^, 1 mL/kg; Virbac, Barcelona, Spain)) by cervical dislocation after overnight fast. All experimental procedures were approved by the UACJ-Biomedical Science Institute Ethics Committee (approval date: 24 October 2012), according to the National legislation on the use of animals for (NOM-062-ZOO-1999) [30] and the National Institutes of Health (NIH) Guide for Care and Use of Laboratory Animals.

### 2.5. Biological Samples

Blood samples were obtained by cardiac puncture during anesthesia, collected in anticoagulant-free tubes and centrifuged at 2000× *g* for 10 min at 4 °C to obtain serum, which was stored at −80 °C until use. Livers were carefully removed, rinsed with sterile PBS, blotted on a filter paper to remove the excess of water, weighed and the hepatosomatic index calculated (HIS = liver weight × 100 × body weight^−1^). Livers were frozen in liquid nitrogen and stored at −80 °C until use.

### 2.6. Serum and Hepatic Lipids

Serum samples were analyzed for TAG, TC and HDL-C as-collected, while hepatic lipids (total fat, TAG and TC) from all 18 samples (6 rats/3 diets) were extracted by the Folch method [31] using ice-cold chloroform: methanol (2:1 *v*/*v*) for 20 min. The Folch method is the most effective method for extracting fatty acids, sterols and steroids from biological samples as compared to other lipid extraction methods [32]. The content of total lipids in hepatic samples was expressed as a percentage (%), while the FA and PST profile of liver fat extracts was performed as for edible oils (Section 2.2), and values were expressed as g (FA) or mg (PST) per 100 g of liver fat. TAG were assayed by the glycerol-phosphate oxidase colorimetric method (TAG liquiColor^®^ GPO-PAP; Stanbio Laboratory, USA), while TC and HDL-C by the cholesterol esterase/oxidase method (liquiColor^®^; Stanbio Laboratory, Boerne, USA) following the manufacturer’s protocol. Lipid values are expressed as mM (serum) and mmol/g (hepatic). All assays were performed in quadruplicates in 96-well microplates.

### 2.7. Gene Expression Analysis

Total RNA was isolated from 100 to 150 mg of each liver sample (*n* = 18; 6 rats/3 diets) using TRI reagent (T9424; Sigma-Aldrich, St. Louis, MO, USA) according to the manufacturer’s instructions. The recovered RNA was treated with RNase-free DNase (Promega, 6PIM610, Madison, WI, USA); its integrity (18S and 28S bands) was evaluated by electrophoresis in 1.0% agarose gels stained with ethidium bromide, and its concentration and purity (260/280 nm ratio >1.8) was evaluated in a Quawell Q3000 UV spectrophotometer (Quawell Technology, Inc., San Jose, CA, USA). Each DNase-treated RNA (2 µg; triplicates) was reverse transcribed (RT) to complementary DNA (cDNA) using the GoScript™ reverse transcription system (A5001; Promega) in a MultiGene^TM^ OptiMax Thermal Cycler (Lab Net International, Inc., Edison, NJ, USA).

One hundred nanograms of each cDNA were further amplified by PCR using the GoTaq^®^ green master mix (M7122; Promega). Reaction mixtures were incubated for 5 min at 25 °C, 60 min at 42 °C and 15 min at 70 °C for enzyme inactivation. End point-PCR amplifications proceeded as follows: Cycle 1 (94 °C/120 s), Cycles 2–35 (denaturing (94 °C/30 s)), annealing (*Tm* °C/30 s) and extension (72 °C/40 s). PCR products were stored at −80 °C until analysis. All PCR amplifications, from which the semi-quantitative (relative) gene expression (sqRT-PCR) level was estimated, were always performed under the same analytical conditions, the same cDNA stock and the same Taq DNA polymerase dilution. Gene-specific primers pairs were designed (*Tm* = 57–60 °C) using primer BLAST software from reference sequences deposited in the National Center for Biotechnology Information website (Table 2). Lastly, end point RT-PCR products were separated on 2% agarose gels under 1× TAE buffer, stained with ethidium bromide (0.5 µg/mL in 1× TAE) and visualized using the Protein Simple Red Imager (Protein Simple, Santa Clara, CA, USA). Images were processed and semi-quantified using the ImageJ software (1.47v, WS Rasband-US National Institutes of Health: Bethesda, MD, USA), using 45S pre-rRNA, precursors of 18S, 5.8S and 28S rRNA, as the house-keeping gene (Rn45S; NR_046239.1).

### 2.8. Statistical Analysis

Normally-distributed data (means ± SD) were analyzed by one-way analysis of variance (ANOVA) and the Tukey–Kramer post hoc test to assess differences between groups’ (GSO, CO, CNO) means. Nonparametric variables were evaluated by the Mann–Whitney U test, using the SPSS statistics software 15.0 (SPSS Inc., Chicago, IL, USA). Statistical significance was confirmed as *p* < 0.05.

## 3. Results

### 3.1. Lipid and Antioxidant Profile of Edible Oils

**Figure 1:**
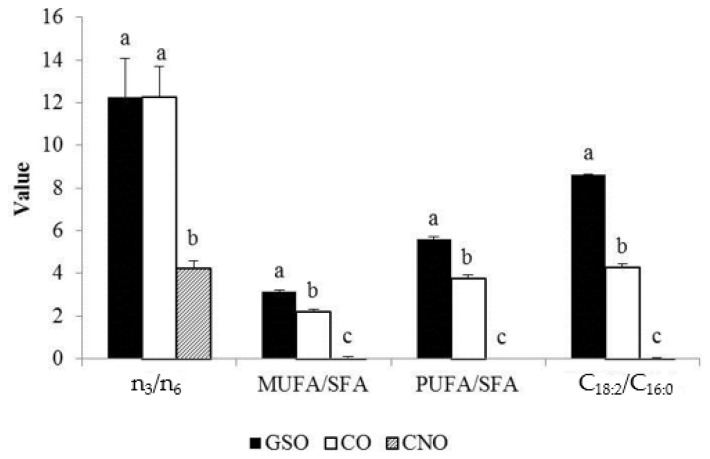
Fatty acid ratios in edible oils. Values are expressed as the mean ± SD ; Different superscripts within the same fatty acid ratio mean statistical differences at *p* ≤ 0.001; saturated (SFA), monounsaturated (MUFA), polyunsaturated (PUFA) fatty acids, fat deterioration index (C_18:2_/C_16:0_).

The FA, PST and AOX profiles significantly differ between oils. CNO differ (*p* ≤ 0.01) from CO and GSO in all thirteen FA reported in Table 3 and ratios in Figure 1.

Individual differences between GSO (higher C_16:0_, C_18:2_) and CO (higher C_18:0_, C_18:3_) were very few (Table 3), but MUFA/SFA, PUFA/SFA and the deterioration index (C_18:2_/C_16:0_) were higher in GSO (*p* ≤ 0.01). Conversely, stigmasterol, β-sitosterol and total PST content was in the order CO > GSO > CNO (*p* ≤ 0.001), and campesterol and ergosterol were only detected in CO (Table 4). Lastly, TP content and radical scavenging capacity (RSC) with the DPPH radical were CO > CNO > GSO and CO > GSO > CNO with the ABTS^2−^ radical (*p* ≤ 0.001; Table 4).

### 3.2. Bioassay Parameters

According to Table 5, cumulative (in 28 days) weight gain (~56 g), food (~257 g) and fat (~25.7 g) intake, FER (~0.22), liver weight (~11 g) and water content (~67.3%) and HSI (~3.6) were not different among feeding groups (*p* ≥ 0.21). However, CNO-fed rats ate 4.9–5.6-times more SFA, but 0.8- and 0.1-times less MUFA and PUFA than GSO or CO fed-rats (*p* ≤ 0.001), leading to a higher deposition of total fats in the liver in CNO-fed rats (~7.0) vs. the other two groups (~5%; *p* < 0.01).

### 3.3. Serum and Hepatic Lipidomic and Antioxidant Response

**Figure 2:**
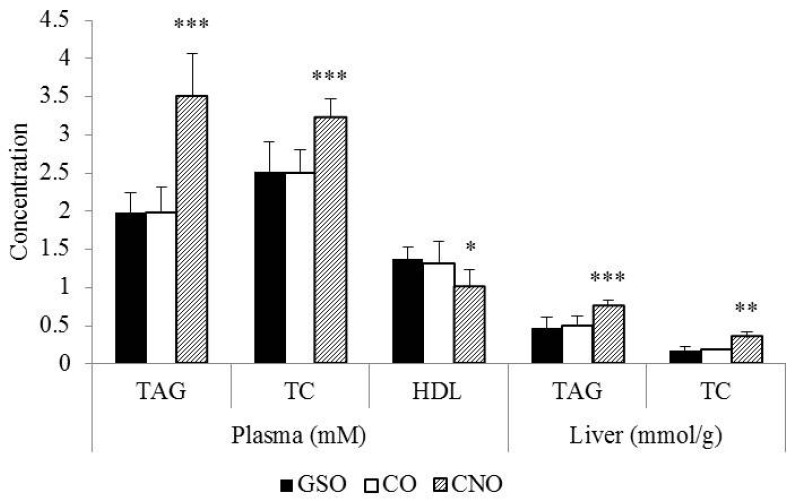
Serum and hepatic lipids. Values are expressed as the mean ± SD (*n* = 6/group) at the 28th day; ^2^ *p* < 0.05 *, *p* < 0.01 **, *p* ≤ 0.001 ***; triacylglycerides (TAG) and total (TC) and high density lipoprotein-cholesterol (HDL).

Serum (mM) and hepatic (mmol/g) TAG (~2.0 and 0.47) and TC (~2.5 and 0.18) responses to GSO and CO were not different (Figure 2), but lower (*p* < 0.01) than those observed in CNO-fed rats (TC = 3.2 and 0.35; TAG = 3.5 and 0.75). Serum HDL-C (mM) was lower (1.0) in the CNO-fed group (*p* < 0.05) as compared to GSO or CO-fed rats (~1.3).

Except for a slightly higher accumulation of margaric acid (C_17:0_, +0.04 g/100 g liver fat) and total SFA (+1.97 g/100 g liver fat) in CO-fed rats, the liver accumulation of all other FA was quite similar to GSO-fed rats (Table 6), consistent with the same total liver fat accumulation (Table 5). Furthermore, CNO-fed rats accumulated more SFA (+10 g/100 g liver fat) and MUFA (+8.5 g/100 g liver fat), but less PUFA (−18 g/100 g liver fat) than CO- and GSO-fed rats. Nevertheless, all groups did not differ on hepatic accumulation of the following FA (Appendix A): SFA (C_8:0_, C_10:0_, C_15:0_, C_18:0_, C_20:0_; *p* ≥ 0.11), MUFA (C_15:1_, C_17:1_, C_20:1_; *p* ≥ 0.57) and PUFA (C_18:2n3_, C_22:5n3_; *p* ≥ 0.19). Lastly, with a few exceptions, PST accumulation was CO > GSO or CNO (Table 7), but serum TAC (FRAP) did not differ (~0.47 mM TE; *p* = 0.08) between diets (Appendix A).

### 3.4. Expression of HDL-Metabolism Related Genes

**Figure 3:**
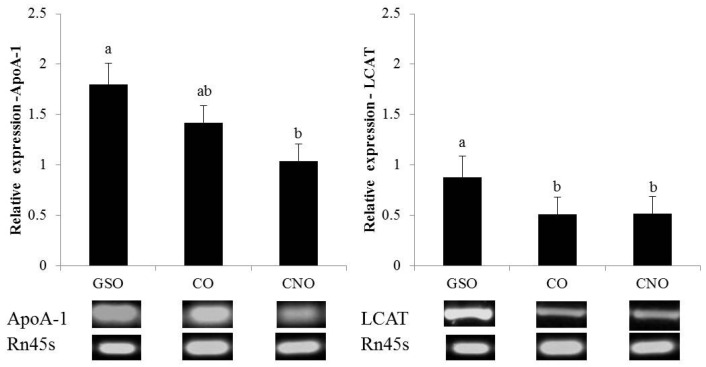

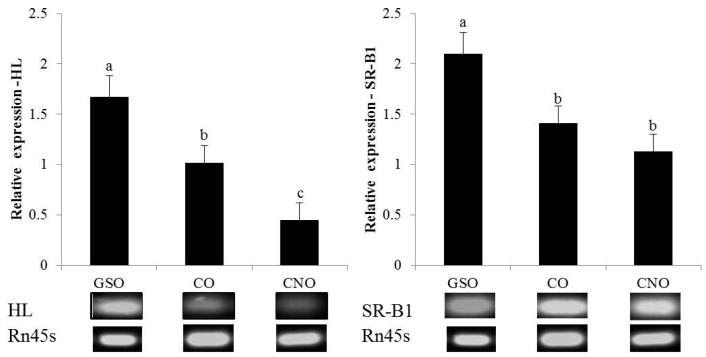
Relative mRNA level of four participants in HDL metabolism. Relative expression normalized to *Rattus norvegicus* 45S pre-ribosomal RNA (Rn45s); data are expressed as the mean ± SD (*n* = 6 rats/group) at the 28th day; Different superscript letters for a same gene means tatistical differences at ≤0.01; lecithin-cholesterol acyltransferase (LCAT), apolipoprotein A1 (ApoA-1), hepatic lipase (HL) and scavenger receptor class B, type 1 (SR-B1).

The sqRT-PCR evaluation revealed that mRNA levels (normalized to Rn45s) of lecithin-cholesterol acyltransferase (LCAT), hepatic lipase (HL) and scavenger receptor class B type 1 (SR-B1), but not that of apolipoprotein A1 (ApoA-1), were upregulated to a higher extent in GSO-fed rats as compared to CO- or CNO-fed rats (Figure 3). Furthermore, the HL lipase mRNA level was higher in CO than CNO-fed rats.

## 4. Discussion

The health benefits of unsaturated FA (MUFA, PUFA) have prompted the new market of specialty and functional oils. This niche market has emerged from the fact that nutrition conscious consumers are seeking novel food products to preserve their health and to prevent severe illness, such as CVD. In this regard, several public organizations such as the American Heart Association recommend replacing SFA for MUFA or PUFA in the daily diet [5]. However, more recently, recommendations have been focused on improving the functionality of circulating HDL particles (e.g., improving reverse-cholesterol transport (RCT) and or anti-inflammatory actions) through pharmacological and dietary strategies [33], since HDL is a potent AOX, anti-inflammatory, antithrombotic and vasodilator agent, besides its well-known role in RCT [34]. From a primary prevention standpoint, non-pharmacological strategies, including small dietary changes, may reduce the cost of treatment (secondary prevention) while promoting a healthy lifestyle in at-risk populations [1,3,4,5]. In this study, three specialty oils differing in their FA, PST and AOX profile were tested for their ability to improve the physiological and genomic response associated with certain aspects of TAG, TC and HDL metabolism, trying to disentangle their overall lipidomic effect in healthy rats.

In countries with a well-established regulation for functional foods, in-market differentiation of specialty oils is based on their specific FA, PST and AOX profile when compared to conventional edible oils, in order to sustain ingredient-based claims. In this sense, all edible oils evaluated in this study (GSO, CO and CNO) qualify as functional oils [8,15,16]. GSO is rich (>85%) in oleic (C_18:1_) + linoleic (C_18:2_) FA (arranged mostly as trilinoleil (43%) and dilinoleil-oleil (23%) TAG), PST (~2.6 mg/g, mainly β-sitosterol), but a moderate source of TP (2.9–36.0 mgGAE/100 g) [15,35,36,37] and RSC (0.33–0.49 mM against ABTS^2−^) [38], and our results are consistent with this evidence (C_18:1_ + C_18:2_ (89.2%), total PST (1.72 mg/g), β-sitosterol (1.52 mg/g), TP (2.4 mgGAE/100 g) and RSC (0.38 mM against ABTS^2−^)). GSO is also a good source of lipophilic antioxidants, such as α, γ-tocopherols and α, γ-tocotrienols [15,36], although they were not evaluated in this study. The CO profile was very similar to other CO previously reported, at least in its C_16:0_ + C_18:1_ + C_18:2_ (>85 g/100 g) and PST (11 mg/g) content [39,40]. The FA profile of CNO was almost identical to other CNO [15,40], although its TP and PST content was lower than previously reported [40,41]. Nevertheless, lauric acid (C_12:0_) from coconut oil (CNO) may exert a TC-lowering effect [8]; α-linolenic acid (C_18:3_) + PST + TP in CO may reduce several risk factors for atherosclerosis [20], inflammation [18] and dyslipidemia [42]; and the PUFA/SFA ratio in GSO may reduce many risk factors for CVD [12,13,14,15].

It is noteworthy that CO showed a higher content of stearic acid (C_18:0_), α-linolenic acid (C_18:3_), total/specific PST, TP and RSC, a similar *n*3/*n*6 ratio, but a lower PUFA/SFA ratio than GSO, while CNO had the highest SFA content (92%, 73.8% from C_12:0_ and C_14:0_) and a negligible content of PST and TP as compared to GSO and CO. The higher deteriorating index in GSO (8.6) as compared to CO (4.3) indicates that the former is more susceptible to oxidation, and so, it is not suitable for frying [36,43]. However, the quantity (CO > GSO > CNO) and chemical nature of AOX species (TP, tocols and PST) in these specialty oils may confer them a higher shelf life, although this was not evaluated in this study.

Health claims related to CVD prevention require a rigorous scientific sustentation of all (negative and positive) physiological effects after the intake of functional edible oils. These evaluations should be performed in convenient rodent models and/or human intervention trials in order to stablish significant associations between their daily intake and certain physiological outcomes, such as a lower risk for oxidative stress, inflammation, hypertension or dyslipidemia [18,19,20,42]. In this study, the lipidomic and antioxidant response to GSO, CO and CNO after a sub-chronic ad libitum intake (28 days) of moderate fat diets (10% *w*/*w*; 23% energy) in healthy young Wistar rats was studied. This protocol was used with the intention to develop a low degree of metabolic disturbances due to a +5% (*w*/*w*) fat intake above the recommendation for Wistar rats [29,39], but not a marked steatosis [44], a higher body fat, dyslipidemia and leptinemia [17] or a higher visceral fat and hyperinsulinemia [39], which are commonly observed in young Wistar rats fed high fat (≥20% *w*/*w*; ≥50% energy from fat) diets. Furthermore, GSO and CO were selected intentionally due their similar FA profile [15,38,40] and possibly different PST and AOX content; also, a high SFA- and cholesterol-free oil (CNO) with negligible PST or AOX content served as control diet, since isoenergetic SFA-rich diets are more obesogenic than PUFA-rich diets [39,45].

In this study, most bioassay parameters did not change among experimental diets at the 28th day, including weight gain (46–62 g), food intake (230–287 g), FER (0.20–0.24) and HSI (3.3%–3.9%). Alsaif et al. [39] fed male Wistar rats (150–160 g) with 10% fat diets (prepared with CO, olive oil or low fat butter oil) for 35 days observing weight gains between 76 and 80 g and HSI ~3.0% with no apparent differences between edible oils. Dominguez-Avila et al. [17] fed male rats (~148 g) with a 10% fat diet (5% lard/5.0% corn oils) for 63 days observing a weight gain of 271.3 g and an FER of 0.20, while Fakhoury-Sayegh et al. [44] fed male rats (150–180 g) with a 15.2% fat-diet (7% soya/8.2% butter oils; 407.5 kcal/100 g; 31.5% energy from fat) for 112 days observing a weight gain of 229 g and an HSI of 2.1%. Together, all of these studies suggest a time + fat quantity effect on weight gain, but not in FER or HIS, not necessarily associated with a certain type of edible oil at least for protocols with sub-chronic fat consumption such as these; it is noteworthy that the authors did not observed steatohepatitis in their moderate fat-fed rat model.

Furthermore, Hurtado de Catalfo et al. [21] fed male Wistar pups (48 ± 3 g) for 60 days with normal-fat diets (7% *w*/*w*; 17% energy from fat) prepared with CNO (high SFA), olive oil (high MUFA), soybean oil and GSO (different PUFA/SFA), demonstrating a higher rate of body weight gain and FER in CNO-fed rats as compared to all other diets, so an age-related effect on bioassay parameters can be also assumed. Lastly, significant differences (*p* ≤ 0.001) on SFA, MUFA and PUFA intakes were observed between diets, which in turn may cause other metabolic derangements, such as dyslipidemia, endothelial dysfunction or higher adiposity (total body, abdominal and hepatic) as those seen with a sub-chronic intake of high fat [17,39,44] or oil-fried (12.3% *w*/*w*, 28% energy canola oil (MUFA/SFA = 1.5, PUFA/SFA = 3.9) + peroxynitrites) diets [46].

The effect of reducing and/or changing dietary fat sources instead of modifying other macronutrients in the daily diet (e.g., dietary fiber and protein) seems to be a more promising way to prevent CVD [4,9]. Particularly, dietary fats are well known to affect TAG and cholesterol homeostasis. A lesser SFA intake with no other dietary modification reduces LDL-C, HDL-C and TC simultaneously [3,7,9], while human and animal studies have shown that consuming PUFA (particularly, n3) and MUFA, to a lower extent, reduces blood LDL-C, but increases HDL-C levels when compared to SFA [12,40,47]. Here, serum TAG and TC increased, while HDL-C decreased in CNO-fed rats as compared to CO- or GSO-fed rats whose serum lipid response was quite similar; the latter effect may be related to the nature and sn-2 position of FA in TAGs [48] from these edible oils. Moreover, data from Table 5 indicate that CNO-fed rats had a higher intake of SFA and accumulated more fat in liver (new data in this new version of the manuscript) as total cholesterol, triacylglycerides (Figure 2) and SFA (mainly as palmitic acid (C_16:0_); Table 6), which in turn modified the mRNA levels of certain enzymes and proteins involved in reverse-cholesterol transport. The hypercholesterolemic (high TC and LDL-C, low HDL-C) and hypertriglyceridemic effect of CNO has been previously reported in rats [49] and humans [50], despite the fact that its lauric acid (C_12:0_) alone is efficient to lower TC [8]. Thus, our study provides a further argument as to the null effect of coconut oil as a superfood.

However, the effect of GSO vs. other PUFA-rich edible oils on the murine serum lipidome has generated controversial results. Chang et al. [51] postulated that a higher (PUFA + MFA)/SFA ratio increases serum TC, TAG and VLDL-C levels, and so, GSO (ratio 8.72) should behave worse than CO (ratio 5.93) on this matter. Asadi et al. [52] fed young Wistar rats (~225 g) with CO and GSO, besides water and a chow diet, ad libitum for 10 weeks observing the same feed and oil intake, serum TC and HDL-C, but a lower serum LDL-C level in CO-fed rats. Kim et al. [53] observed significant reductions in TC, LDL-C and the atherogenic index, but a higher HDL-C/TC ratio in GSO-fed rats as compared to those fed with lard or soybean oil. De la Torre-Carbot et al. [54] evaluated the serum lipid profile of male Wistar rats fed with different commercial oils, including GSO (10% *w*/*w*; 14.4% energy as added oil) for two weeks, showing that GSO promoted a lower liver weight than soybean oil and higher HDL-C and LDL-C levels as compared to other PUFA-rich edible oils. However, Al-Attar [55] showed higher TC and HDL-C levels in CO- vs. GSO-fed (orally administered 2 g/kg BW) Wistar rats (85–93 g BW), both conferring protection against diazinon-induced hepatotoxicity. Discrepancies between all of these studies, including ours, may rely on the duration and dose used, the rat’s physiological condition and the source of GSO (e.g., commercial vs. fresh cold-pressed).

This study also showed that the hepatic deposition of FA, but mostly PST, was different between specialty oils. CNO-fed rats accumulated more SFA and MUFA, but less PUFA as compared to CO- and GSO-fed rats, but the FA deposition in CO and GSO was quite similar; in fact, C_18:0_ (higher in GSO) and C_18:1cis_ (higher in CO) were equally deposited, while C_17:0_ (same in CO and GSO) and total SFA (higher in CO) were deposited differently. Moreover, the hepatic deposition of PST was higher in CO- than in GSO-fed rats (*p* ≤ 0.001). To our knowledge, the specific hepatic deposition of PST from GSO (1.72 mg/g, mostly β-sitosterol) is reported here for the first time.

The effect of GSO’s lipids, other than PST, on serum, hepatic and extrahepatic lipidomes has been studied by others. Hurtado de Catalfo et al. [21] reported that at the end of the feeding period (60 days), CNO, olive and soybean oil and GSO differently modified the hepatic lipid composition; Lastly, Shinagawa et al. [56] evaluated the effect of intra-gastric administration (3 and 6 mL/kg) of a cold-pressed GSO or a soybean oil in male Wistar rats (~54 g BW) for 65 days observing a dose-response effect on the weight of retroperitoneal fat and liver, independently of the administered oil; despite the fact that both edible oils deposited their total SFA and MUFA in liver similarly, differences in PUFA deposition in adipose tissue (retroperitoneal) were observed, particularly a higher C_18:3n3_ deposition in soybean oil-fed rats as compared to GSO. Since liver weight (11.7 g vs. 11.4 g) and water content (66.7% vs. 67.9%; 7.8 g and 7.4 g) were similar between GSO- and CO-fed rats, our results suggest that hepatic FA handling instead of their total deposition as TAG (~3.9 g) could be different. This in turn may affect several metabolic routes, including the regulatory mechanisms related to hepatic FA and cholesterol homeostasis [17,57,58], while a different hepatic deposition of PST may have affected different signaling pathways involved in liver inflammation [18,20] and lipoprotein trafficking [42].

The liver plays a critical role in controlling FA and cholesterol homeostasis [17,34,58] in full integration with peripheral tissues, such as small bowel [34,42,58], muscle [52] and adipose tissue [46,56]. The epigenetic role of FA on the hepatic metabolism of TAG and of PST on cholesterol metabolism has been the traditional approach, although few studies address the communication between these two metabolic pathways. A failure to handle FA may result in hepatic steatosis as that seen with high fat (particularly SFA) and hypercholesterolemic diets, increasing the risk for vascular degeneration, hypertension and insulin resistance [17,39,44] and lowering serum HDL-C levels while increasing LDL-C. This explains the inverse relationship between HDL-C serum levels and the risk for CVD, since HDL-C reflects the body’s capacity to return cholesterol from peripheral organs to the liver (also known as RCT) [58] and an efficient enzymatic machinery to convert SFA into MUFA or PUFA under normal conditions [9]. Moreover, because this lipoprotein is also involved in AOX mechanisms [34], pharmaceutical interventions aimed to raise HDL-C levels and/or to increase its functionality (e.g., improving RCT and or anti-inflammatory actions) rather than reducing LDL-C, seems to be the best approach to prevent and/or treat CVD [33].

However, HDL-C metabolism is more complex than that of LDL-C, since many proteins, mostly regulated at the transcriptional level, participate in HDL remodeling, its hepatic clearance and RCT [57,58]. TAG (from dietary FA) and other lipids (including PST and dietary cholesterol) are packed into chylomicrons by intestinal cells, which travel up to the liver [34]. Free cholesterol within cells generates oxysterols, which act as ligands for nuclear liver receptors (LXR) and retinoid receptors (RXR), an action that could be reversed by MUFA + PUFA [17] and PST. HDL particles are packed in plasma, in the extravascular space, the liver and small bowel are involved in nascent HDL synthesis providing ApoA-1 (both) or ApoA-2 (just liver), but also in RCT [58]. RCT is mediated by a basic interaction between the ATP-binding cassette (ABC) transporter-A1 (in intestine and peripheral tissues) and ApoA-1 (in HDL) [59]. TAG-rich remnant lipoproteins are converted to LDL-C, which may interact with its receptor (LDLr) in liver and peripheral tissues and/or interchange its TAG content for cholesteryl esters from HDL particles and vice versa by means of cholesteryl ester transfer protein (CETP), whose expression is regulated by LXR/RXR. HDL_3_ (dense, rich in esterified cholesterol) is further metabolized into HDL_2_ (less dense, rich in phospholipids) by LCAT and phospholipid transfer protein (PLTP) in a concerted action with HL (synthesized and secreted by hepatocytes and macrophages), which performs the same reaction in the opposite direction. Lastly, HDL_2_ (marker of acute myocardial infarction) is hydrolyzed by HL, and its components enter the liver via SR-B1, which removes esterified cholesterol from the liver, which is excreted in bile.

Lastly, our sqRT-PCR assay indicates that HL, LCAT and SR-B1, but not ApoA-1 mRNA levels increased in GSO-fed rats as compared to CO or CNO groups (*p* ≤ 0.01). Kim et al. [53] found a lower epididymal, but not hepatic fat in GSO-fed rats as compared to soybean oil or lard, attributing this effect to a concerted action of C_18:2_ (58%–78%), tocotrienols (4.5–5.3 mg/g oil) and TP (0.10–0.34 mg/g) contents in GSO. GSO and CO assayed in this study promoted the same HDL-C serum level, but their TP and PST content differ (higher in CO); so, a more efficient hepatic FA handling, but not serum AOX response, nor PST hepatic deposition, seemed to drive the effects on the expression of these hepatic genes. This hypothesis is based on the fact that a concurrent ApoA-1 (same in CO and GSO) and ABCA1 expression prevents hepatic steatosis by stimulating a lower deposition of FA (as TAG) and TC, while suppressing FA synthesis by reducing 27-hydroxyesterol levels [59]; this effect plus a higher expression of RCT-involved enzymes/proteins in (SR-B1) and out (LCAT/HL) the liver may stimulate a more efficient FA and TC mobilization.

## 5. Conclusions

Novel insights into the study of HDL metabolism indicate that we are at the beginning of a new line of research on dysfunctional HDL-C (dysHDL-C), although many issues need to be considered before confirming its predictive role of CVD [33,34]. For instance, the RCT-improving and anti-inflammatory effects of HDL-C are emerging subjects of research [57,58,59,60]. In this sense, our study attempts to show that the hepatic fatty acid handling, but not antioxidant response, nor hepatic phytosterol deposition, could be related to a more efficient reverse-cholesterol transport in GSO-fed rats as compared to CO or CNO.

The authors recognize that this study has several limitations. Major drawbacks are the lack of HPLC sub-fractionation of serum lipoproteins (VLDL/LDL/IDL/HDL) at the end of the study, the fact that we only measured total and HDL-cholesterol, not considering more efficient and standardized ways to evaluate HDL functionality [57,58,59,60], and the failure to specifically link the mRNA expression of four genes related to RCT with the fatty acid and phytosterol deposition and related antioxidant microenvironment in the liver. Particularly, measuring the in vivo function of HDL could be of significant importance to test the effectiveness of different RCT-enhancing diet therapies [60]. The lipidomic approach used in this study (circulating lipids, lipid deposition in liver and gene expression) just provide a little bit of evidence as to the possibility of a more efficient reverse-cholesterol transport (RCT); however, in order to support this evidence, fatty acid and phytosterol tracer studies and/or sub-chronic feeding studies using knockout rodent models should be performed in the near future to get a better understanding of the health benefits of consuming GSO over other specialty oils.

## Figures and Tables

**Table 1:** Experimental diets (g/100 g).

Ingredient	GSO	CO	CNO	Ingredient	GSO	CO	CNO
GSO ^1^	10.0			DL-methionine ^2,4^	0.2	0.2	0.2
CO ^1^		10.0		Cellulose ^2^	5.0	5.0	5.0
CNO ^1^			10.0	AIN-93G-Mineral mix ^2^	3.5	3.5	3.5
*SFA*	1.1	1.4	9.2	AIN-93-Vitamin mix ^2^	1.0	1.0	1.0
*MUFA*	3.2	3.2	0.7	Choline chloride ^5^	0.02	0.02	0.02
*PUFA*	5.7	5.4	0.1	Sucrose + maltodextrins ^1^	17.6	17.7	17.7
Casein ^2,3^	21.0	21.0	21.0	Corn starch ^1^	38.7	38.8	38.8

^1^ Food-grade (several trademarks); grapeseed oil (GSO), corn oil (CO), coconut oil (CNO); ^2^ food-grade from Bioserv, Inc. (Frenchtown, NJ, USA); ^3^ ANRC (American National Research Council) grade: 95% protein, vitamin-free, 2.5 of total sulfur amino acids (TSA)/100 g protein; ^4^ to reach >0.98 g/100 g diet of TSA [29]; ^5^ 99% pure (74.6% choline); saturated (SFA), mono- (MUFA) and poly-unsaturated fatty acids (PUFA). All diets were isocaloric (4 kcal/g).

**Table 2:** PCR primers.

Gene	NCBI-RS	Protein	Primer Pair (5′-3′)	*Tm* °C
*Rn45s*	NR_046239.1		Fw: GTTCCGCTCACACCTCAGAT Rv: CAAGTGCGTTCGAAGTGTCG	58
*Lcat*	NM_017024.2	LCAT	Fw: ACACAGGCCAAGACTTCGAG Rv: GGTTGGGGACTTAGGAGTGC	56
*ApoA1*	NM_012738.1	ApoA-1	Fw: CCTGGACAACTGGGACACTC Rv: GCCCAGAACTCCTGAGTCAC	57
*Lipc*	NM_012597.2	HL	Fw: GCACTATGCTATTGCCGTGC Rv: TTGATGCCCACACTCAGACC	60
*Srb1*	NM_031541.1	SR-B1	Fw: CCCCATGAACTGTTCCGTGA Rv: GATCTTCCCTGTTTGCCCGA	57

National Center for Biotechnology Information (NCBI) reference sequence (RS); genes: *Rattus norvegicus* 45S pre-ribosomal RNA (Rn45s), lecithin cholesterol acyltransferase (Lcat), apolipoprotein A1 (Apoa1), lipase C hepatic type (Lipc) and scavenger receptor class B, member 1 (Scarb1) mRNA. Primer forward (Fw) and reverse (Rv).

**Table 3:** Fatty acid profile in edible oils ^1^.

Fatty Acid	GSO	CO	CNO
SFA	10.24 ± 0.32 ^a^	14.38 ± 0.65 ^c^	92.13 ± 2.02 ^b^
C_6:0_	≤0.001 ^a^	≤0.001 ^a^	0.56 ± 0.04 ^b^
C_8:0_	≤0.001 ^a^	≤0.001 ^a^	6.23 ± 0.10 ^b^
C_10:0_	≤0.001 ^a^	≤0.001 ^a^	5.82 ± 0.02 ^b^
C_12:0_	≤0.001 ^a^	≤0.001 ^a^	42.67 ± 1.42 ^b^
C_14:0_	0.07 ± 0.03 ^a^	0.04 ± 0.02 ^a^	21.12 ± 0.97 ^b^
C_16:0_	6.63 ± 0.11 ^a^	12.47 ± 0.50 ^c^	11.69 ± 0.09 ^b^
C_17:0_ *	0.03 ± 0.02 ^ab^	0.05 ± 0.02 ^a^	≤0.001 ^b^
C_18:0_	3.49 ± 0.22 ^a^	1.79 ± 0.12 ^c^	4.03 ± 0.06 ^b^
MUFA	32.09 ± 0.99 ^a^	31.60 ± 0.52 ^a^	6.63 ± 0.10 ^b^
C_14:1_	0.03 ± 0.02 ^a^	0.02 ± 0.01 ^a^	0.13 ± 0.06 ^b^
C_16:1_ **	0.09 ± 0.02 ^a^	0.10 ± 0.05 ^a^	≤0.001 ^b^
C_18:1_	32.00 ± 1.00 ^a^	31.50 ± 0.53 ^a^	6.63 ± 0.10 ^b^
PUFA	57.21 ± 1.06 ^a^	53.65 ± 0.32 ^c^	0.76 ± 0.06 ^b^
C_18:2_	57.20 ± 1.06 ^a^	53.33 ± 0.32 ^c^	0.75 ± 0.05 ^b^
C_18:3_	≤0.001 ^a^	0.32 ± 0.02 ^c^	≤0.001 ^a^

^1^ Values are expressed as the mean ± SD (g/100 g); different superscript letters within the same line mean statistical differences at *p* ≤ 0.001, otherwise specified (*p* < 0.05 *, *p* < 0.01 **).

**Table 4:** Phytosterol and antioxidant profile of edible oils ^1,2^. TE, trolox equivalents.

Variable	GSO	CO	CNO
*Phytosterols* (mg/g)			
Campesterol	≤0.001 ^a^	0.14 ± 0.00 ^b^	≤0.001 ^a^
Ergosterol	≤0.001 ^a^	0.97 ± 0.01 ^b^	≤0.001 ^a^
Stigmasterol	1.20 ± 0.02 ^a^	0.52 ± 0.01 ^b^	≤0.001 ^c^
β-Sitosterol	1.52 ± 0.01 ^a^	8.37 ± 0.04 ^b^	≤0.001 ^c^
Total	1.72 ± 0.01 ^a^	10.0 ± 0.02 ^b^	≤0.001 ^c^
*Antioxidant capacity*			
TP (mgGAE/100 g)	2.36 ± 0.12 ^a^	2.96 ± 0.12 ^b^	2.58 ± 0.14 ^c^
ABTS (mM TE)	0.38 ± 0.01 ^a^	4.58 ± 0.48 ^b^	0.02 ± 0.01 ^c^
DPPH (mM TE)	0.55 ± 0.01 ^a^	24.68 ± 0.94 ^b^	1.95 ± 0.39 ^c^

^1^ Values are expressed as the mean ± standard deviation from six rats per dietary treatment at the end of the experiment; ^2^ Different superscript letters within the same line mean statistical differences (*p* ≤ 0.001); total phenolic compounds (TP), radical scavenging capacity (ABTS or DPPH).

**Table 5:** Bioassay parameters ^1,2,3^.

Variable	GSO	CO	CNO
Initial body weight	303.8 ± 26.8	298.2 ± 30.0	309.7 ± 24.7
Final body weight	365.8 ± 20.1	358.5 ± 29.1	356.3 ± 27.8
Weight gain	62.0 ± 21.0	60.0 ± 7.2	46.0 ± 11.0
Total food intake	287.2 ± 52.5	254.4 ± 34.3	229.8 ± 37.4
Total fat intake	28.7 ± 5.3	25.4 ± 3.4	23.0 ± 3.7
SFA intake ***	3.2 ± 0.6 ^a^	3.6 ± 0.5 ^a^	21.1 ± 3.4 ^b^
MUFA intake ***	9.2 ± 1.7 ^a^	8.1 ± 1.1 ^a^	1.6 ± 0.3 ^b^
PUFA intake ***	16.4 ± 3.0 ^a^	13.7 ± 1.9 ^a^	0.2 ± 0.0 ^b^
FER	0.22 ± 0.08	0.24 ± 0.03	0.20 ± 0.05
Liver weight	11.7 ± 1.0	11.4 ± 0.8	10.2 ± 2.1
HSI (%)	3.9 ± 0.4	3.8 ± 0.2	3.3 ± 0.7
Liver water (%)	66.7 ± 1.8	67.9 ± 1.2	67.4 ± 0.3
Liver fat (%) **	4.9 ± 1.0 ^a^	5.0 ± 0.7 ^a^	6.9 ± 1.0 ^b^

^1^ Values are expressed as the mean (g) ± standard deviation from six rats per dietary treatment accumulated at the 28th day, otherwise specified; ^2^ Different superscript letter in the same line mean statistical differences (*** *p* ≤ 0.001, ** *p* ≤ 0.01); ^3^ food efficiency ratio (FER = weight gain (g)/diet consumed (g)), hepatosomatic index (HSI = liver weight × 100 × body weight^−1^), saturated (SFA), monounsaturated (MUFA), polyunsaturated (PUFA) fatty acids.

**Table 6:** Fatty acid deposition in liver ^1^.

Fatty Acid	GSO	CO	CNO
SFA	31.28 ± 3.67 ^a^	33.25 ± 2.08 ^b^	42.29 ± 1.87 ^c^
C_12:0_	0.11 ± 0.03 ^a^	0.08 ± 0.04 ^a^	1.20 ± 0.42 ^b^
C_14:0_	0.38 ± 0.05 ^a^	0.46 ± 0.10 ^a^	2.81 ± 0.33 ^b^
C_16:0_	16.36 ± 0.80 ^a^	18.35 ± 0.57 ^a^	23.19 ± 1.08 ^b^
C_17:0_	0.34 ± 0.06 ^a^	0.38 ± 0.04 ^b^	0.21 ± 0.05 ^c^
C_22:0_	0.17 ± 0.05 ^a,c^	0.16 ± 0.03 ^a^	0.26 ± 0.02 ^b^
MUFA	13.91 ± 2.73 ^a^	15.55 ± 1.93 ^a^	23.31 ± 2.65 ^b^
C_16:1_	0.88 ± 0.41 ^a^	0.88 ± 0.18 ^a^	4.03 ± 1.30 ^b^
C_18:1cis_	11.82 ± 2.29 ^a^	13.63 ± 1.81 ^a^	18.12 ± 1.48 ^b^
PUFA	53.16 ± 1.37 ^a^	51.27 ± 0.75 ^a^	34.41 ± 2.52 ^b^
C_18:2cis_	25.08 ± 1.11 ^a^	23.35 ± 2.16 ^a^	10.38 ± 1.37 ^b^
C_18:3n6_ **	0.30 ± 0.11 ^a^	0.34 ± 0.11 ^a^	0.16 ± 0.03 ^a^
C_20:2_ **	0.85 ± 0.12 ^a^	0.78 ± 0.05 ^ab^	0.57 ± 0.23 ^b^
C_20:3n6_	0.56 ± 0.09 ^a^	0.83 ± 0.25 ^a^	1.39 ± 0.16 ^b^
C_20:4n6_	22.13 ± 2.09 ^a^	21.29 ± 1.91 ^a^	15.20 ± 1.33 ^b^
C_22:4n6_	0.61 ± 0.14 ^a^	0.69 ± 0.09 ^a^	0.27 ± 0.06 ^b^
C_22:6n3_	2.77 ± 0.39 ^a^	2.94 ± 0.47 ^a^	5.54 ± 0.28 ^b^

^1^ Fatty acids were expressed as the mean (g/100 g liver fat) ± standard deviation from six rats per dietary treatment at the end of the experiment (28th day). Different superscript letters within the same line mean statistical differences at *p* ≤ 0.001, otherwise specified (*p* < 0.01 **); saturated (SFA), monounsaturated (MUFA), polyunsaturated (PUFA) fatty acids.

**Table 7:** Phytosterol deposition in liver ^1^.

Phytosterol	GSO	CO	CNO
Campesterol	0.03 ± 0.02 ^a^	0.34 ± 0.05 ^b^	0.13 ± 0.04 ^c^
Ergosterol	0.32 ± 0.18 ^a^	0.75 ± 0.28 ^b^	0.07 ± 0.02 ^a^
Stigmasterol **	0.09 ± 0.05 ^ab^	0.13 ± 0.03 ^a^	0.05 ± 0.03 ^b^
β-Sitosterol	0.52 ± 0.23 ^a^	1.95 ± 0.56 ^b^	0.26 ± 0.09 ^a^
Total	0.96 ± 0.45 ^a^	3.16 ± 0.62 ^b^	0.50 ± 0.18 ^a^

^1^ Values are expressed as the mean (mg/g) ± standard deviation (*n* = 4); Different superscript letters within the same line means statistical differences at *p* ≤ 0.001, otherwise specified (*p* < 0.01 **).

## Appendix A

**Table A1:** Non-modified physiological parameters after GSO, CO and CNO intake ^1,2^.

Variable	GSO	CO	CNO	*p*
*Hepatic FA* (mg/g)				
C_8:0_	0.25 ± 0.04	0.18 ± 0.10	0.16 ± 0.05	0.11
C_10:0_	0.08 ± 0.04	0.06 ± 0.03	0.09 ± 0.01	0.36
C_15:0_	0.22 ± 0.03	0.20 ± 0.02	0.20 ± 0.03	0.49
C_18:0_	14.96 ± 2.37	13.25 ± 1.48	14.14 ± 0.58	0.29
C_20:0_	0.07 ± 0.02	0.11 ± 0.02	0.07 ± 0.07	0.16
C_15:1_	0.13 ± 0.03	0.11 ± 0.02	0.13 ± 0.03	0.57
C_17:1_	0.45 ± 0.16	0.50 ± 0.09	0.44 ± 0.05	0.68
C_20:1_	0.24 ± 0.02	0.27 ± 0.03	0.24 ± 0.12	0.75
C_18:2n3_	0.43 ± 0.12	0.49 ± 0.15	0.34 ± 0.12	0.19
C_22:5n3_	0.39 ± 0.10	0.41 ± 0.10	0.50 ± 0.17	0.32
*Serum TAC*				
FRAP (mM TE)	0.47 ± 0.01	0.48 ± 0.02	0.46 ± 0.01	0.08

^1^ Values are expressed as the mean ± standard deviation, 28-day consumption; ^2^ grapeseed oil (GSO), corn oil (CO), coconut oil (CNO), ferric reducing ability (FRAP), total antioxidant capacity (TAC).
